# Culture-Dependent and Culture-Independent Characterization of the Olive Xylem Microbiota: Effect of Sap Extraction Methods

**DOI:** 10.3389/fpls.2019.01708

**Published:** 2020-01-21

**Authors:** Manuel Anguita-Maeso, Concepción Olivares-García, Carmen Haro, Juan Imperial, Juan A. Navas-Cortés, Blanca B. Landa

**Affiliations:** ^1^ Institute for Sustainable Agriculture, Spanish National Research Council (CSIC), Córdoba, Spain; ^2^ Institute of Agricultural Sciences, Spanish National Research Council (CSIC), Madrid, Spain

**Keywords:** microbiome, xylem, culture, next-generation sequencing, vascular pathogens

## Abstract

Microbial endophytes are well known to protect host plants against pathogens, thus representing a promising strategy for the control of xylem-colonizing pathogens. To date, the vast majority of microbial communities inhabiting the olive xylem are unknown; therefore, this work pursues the characterization of the xylem-limited microbiome and determines whether the culture isolation medium, olive genotype, and the plant material used to analyze it can have an effect on the bacterial populations retrieved. Macerated xylem tissue and xylem sap extracted with the Scholander chamber from olive branches obtained from two cultivated and a wild olive genotypes were analyzed using culture-dependent and -independent approaches. In the culture-dependent approach using four solid culture media, a total of 261 bacterial isolates were identified after performing Sanger sequencing of 16S rRNA. Culturable bacteria clustered into 34 genera, with some effect of culture media for bacterial isolation. The cultivated bacteria belonged to four phyla and the most abundant genera included *Frigoribacterium* (18.8%), *Methylobacterium* (16.4%), and *Sphingomonas* (14.6%). On the other hand, in the culture-independent approach conducted using Illumina MiSeq 16S rRNA amplicon sequencing [next-generation sequencing (NGS)] of the xylem extracts, we identified a total of 48 operational taxonomic units (OTUs) belonging to five phyla, being *Sphingomonas* (30.1%)*, Hymenobacter* (24.1%) and *Methylobacterium* (22.4%) the most representative genera (>76% of reads). In addition, the results indicated significant differences in the bacterial communities detected in the xylem sap depending on the genotype of the olive tree studied and, to a minor extent, on the type of sap extraction method used. Among the total genera identified using NGS, 14 (41.2%) were recovered in the culture collection, whereas 20 (58.8%) in the culture collection were not captured by the NGS approach. Some of the xylem-inhabiting bacteria isolated are known biocontrol agents of plant pathogens, whereas for others little information is known and are first reported for olive. Consequently, the potential role of these bacteria in conferring olive tree protection against xylem pathogens should be explored in future research.

## Introduction

Olive tree (*Olea europaea* L.) is one of the oldest cultivated trees and has been part of traditional Mediterranean agriculture ever since Roman times. Totaling over 2,000 cultivars (Lavee, 1990), the culture spread from Asia along Syria, Iran, and Palestine to the rest of the Mediterranean basin *ca.* 6,000 years ago. Since then, olive oil has been a food staple of Mediterranean countries (Müller et al., 2015). Olive oil is considered a high-quality food with multiple beneficial effects for human health, mainly due to its high content in polyphenols, which has induced an increase in olive oil demand and trade worldwide (Landa et al., 2019). Nowadays, world olive cultivation is estimated to cover 10.8 million ha, which results in an olive oil production of about 3.05 million tons, of which *ca.* 9.5 million ha of olives are grown in the Mediterranean region, accounting for 95% of the cultivated olive area worldwide (i.e., about 98% of the olive oil and 80% of table olive production are form Mediterranean countries) (IOC, www.internationaloliveoil.org/; FAOSTATS, http://www.fao.org/faostat/). Olive crop is of great value within the Mediterranean Basin, being critical for the sustainment and maintenance of Mediterranean ecosystems as an integral part of their landscape. Moreover, olive crop is particularly well adapted to cultivation in less accessible areas, including mountain slopes and hillsides, where it helps control soil erosion by reducing the surface runoff of soil and increases soil fertility and nutrient retention (Gómez et al., 2014). Finally, as a major landscape player, it contributes to the establishment of ecological niches for different organisms, thus helping in the maintenance of biodiversity (Rey, 2011). Consequently, since olive is a “high natural value” agricultural system with relevant economic and environmental roles, representing an important part of the heritage and sociocultural life across the Mediterranean, its cultivation should be maintained and preserved.

Nowadays, the health of the olive groves is being seriously threatened, as consequence of a notable increase, both in extent and in severity, of diseases caused by various pathogens, which are capable of adversely affect their growth and production. Among olive diseases, those caused by the vascular plant pathogenic bacterium *Xylella fastidiosa* (specifically from subspecies *multiplex* and *pauca*) and the soilborne vascular fungus *Verticillium dahliae* are, without a doubt, global threats for olive production worldwide (Jiménez-Díaz et al., 2011; Saponari et al., 2018; Almeida et al., 2019; Landa et al., 2019; Landa et al., 2020). Xylem vessels are considered ideal niches for microbial endophytes (bacteria or fungi; both beneficial or pathogenic) by providing an effective internal pathway for dispersion throughout the plant and a continuous source of nutrients (McCully, 2001). However, despite significant progress in research on plant microbiota over past years, only a few number of publications have revealed the nature and role of the xylem microbiome, and its relationship to plant health and crop productivity (e.g., Deyett et al., 2017; Fausto et al., 2018; Deyett and Rolshausen, 2019). This fact may be due to microbiome complexity, technical difficulties in the isolation of the xylem-inhabiting microorganisms, as well as to the myriad of biotic and abiotic factors that may affect and determine the microbial community composition and their interaction within the host plant (Vandenkoornhuyse et al., 2015).

With olive, most microbiome studies have focused on determining the microbial composition of the rhizosphere (Mercado-Blanco et al., 2004; Berg and Hallmann, 2006; Mendes et al., 2007; Aranda et al., 2011; Prieto et al., 2011; Montes-Borrego et al., 2013; Caliz et al., 2015; Gómez-Lama Cabanás et al., 2018), whereas only a few studies have focused on the endosphere or xylem microbiomes (Müller et al., 2015; Fausto et al., 2018; Sofo et al., 2019). Unraveling the microbiome of the olive xylem sap should provide a better understanding of the microbes that systemically move throughout the plant or are horizontally transmitted from plant to plant during vegetative propagation. Combined use of culture-dependent and culture-independent approaches such as next-generation sequencing (NGS) technologies may provide a better characterization of the xylem microbiome composition (Turner et al., 2013; Berg et al., 2014) than that obtained when using each approach independently. An essential, parallel step would be to determine the proportion of the microbiome that can be easily isolated and cultured, in order to be subsequently tested and potentially exploited as biocontrol agents of the xylem-inhabiting plant pathogens, such as *X. fastidiosa* or *V. dahliae.* These resident microorganisms could act through direct inhibition or through niche displacement of those pathogens, as commensal and symbiotic organisms that can support the olive immune system, and/or as plant growth promoting agents.

Our research was designed to address this gap in the knowledge by characterizing the bacterial taxa that shape the olive xylem sap microbial communities by using culture-dependent and culture-independent approaches. We also determined in which extent the culture isolation media and the type of plant extract or olive genotype used to isolate the xylem sap bacterial microbiome have an effect on the characterization of its composition, diversity, and structure. Knowledge of the effect of those factors may be essential to identify, isolate, and establish a bacterial collection that might be used subsequently as biocontrol agents against xylem-inhabiting plant pathogens.

## Material and Methods

### Sampling of Olive Trees

Branches (*ca.* 35 cm long, 2 years old) from 8-year-old cultivated olive (*O. europaea* var. *europaea*) trees of “Picual” and “Arbequina” cultivars were sampled from an experimental rainfed field located at the Institute for Sustainable Agriculture from the Spanish National Research Council (IAS-CSIC) in Córdoba (Southern Spain). Trees of both varieties have been submitted to the same agricultural practices throughout the years. Cv. Picual has been found to be highly susceptible to Defoliating (D) and susceptible to Nondefoliating (ND) *V. dahliae* isolates, respectively, while cv. Arbequina has been shown to be susceptible to D and moderately resistant to ND *V. dahliae* (Calderon et al., 2014). In addition, clones of a wild olive tree of *O. europaea* var. *sylvestris* “Acebuche” known to be moderately resistant to D *V. dahliae* infection from a collection of wild-olive genotypes at IAS-CSIC was also sampled. Three 35-cm-long terminal branches (one per tree) from independent “Picual” and “Arbequina” cultivated olive trees and from clone trees of “Acebuche” were sampled in December 2018 to perform xylem sap extraction with the Scholander chamber. Xylem sap was extracted at the end of Autumn, a season of the year when the olive stem water potential is more stable (Iniesta et al., 2009) and lower than 40 bar of pressure, the maximum pressure allowed by the Scholander chamber device that was used to extract the xylem sap (see below). Likewise, for wood chips collection, a similar number of branches (one branch per tree, with three trees in total per olive genotype) were sampled, and 6-cm-long stem portions were selected from each branch (three pieces per branch). All pruned branches were placed in sterile plastic bags, sprayed with distilled water and kept in a cold room at 4°C to avoid desiccation until later processing in the same day.

### Microbiome Extraction From Xylem

The three 6-cm-long pieces of branches from each sample were debarked with a sterile scalpel. Bark tissue or xylem chips were obtained by scraping the debarked woody pieces with a sterile scalpel. A total of 0.5 g of xylem chips was weighted by mixing the chips obtained from all pieces sampled from same branch and tree and placed in a Bioreba bag containing 5 ml of sterile phosphate-buffered saline (PBS). Bioreba bags were closed with a thermal sealer and the content was homogenized with a hand homogenizer (BIOREBA, Reinach, Switzerland). Extracts were stored at 4°C until plating onto culture media, and then at -80°C, until DNA extraction. A total of three replicates per olive tree within each genotype was processed. All the processes described above took place under sterile conditions within a flow hood chamber.

Xylem sap extraction from olive branches was performed with a Scholander pressure chamber connected to a nitrogen cylinder following the Bollard process described by Alexou and Peuke (2013). An external port allowed switching from the internal Scholander chamber to an external 60-cm-long super chamber admitting a maximum of 40 bar of pressure. After inserting the branch in the super chamber, 5 cm of the main stem protruded to the exterior of the lid. At this point, it was important to preserve the branch with no cuts or loose leaves that could compromise the pressure within the chamber. To avoid microbial contamination of the xylem sap from bark and phloem, 2 cm of the main stem was debarked and disinfested. The edge between bark and xylem tissue was covered with parafilm to avoid leaks, and the pressure was increased gradually until xylem sap drops were observed to a maximum of 35 bars. The first drops of xylem sap were discarded to avoid external contamination. Xylem sap was collected within a 15 ml sterile falcon tube placed on ice. An average of 10 ml of xylem sap per genotype and branch was obtained and was preserved at 4°C until plating onto culture media, and then kept at -80°C until DNA extraction. All the processes described above took place under sterile conditions within a flow hood chamber.

### Culture-Dependent Characterization of Xylem Bacteria

For the culture-dependent approach, four solid culture media were evaluated: BCYE (Wells et al., 1981), PD2 (Davis et al., 1981), R2A (Reasoner and Geldreich, 1985), and Nutrient agar (NA) (CONDALAB, Madrid, Spain). R2A and NA are general culture media of low- or high-nutrient contents, respectively. BCYE and PD2 are media specifically designed for isolation of the fastidious bacterium *X. fastidiosa* from xylem tissues.

Aliquots or 1/10 dilutions (100 µl each) of xylem extracts obtained by each of the two techniques indicated above were plated directly onto three plates of each medium and incubated at 28°C in the dark for 2 weeks. After incubation, all bacterial colonies were counted and a representative number of colonies from each medium and genotype was selected based on abundance and colony feature morphological criteria. Selected colonies were purified by triple serial colony isolation in the same medium. Purified isolates were grown at 28°C in the dark for 1 to 2 weeks depending on their rate of growth prior to DNA extraction.

DNA was extracted from a total of 261 bacterial isolates using the DNeasy kit (QIAGEN, Madrid, Spain). The near-complete 16S rDNA gene was amplified using primers 8f (5′-AGAGTTTGATCCTGGCTCAG-3′) and 1492r (5′-ACGGCTACCTTGTTACGACTT-3′) (Weisburg et al., 1991) as described in Aranda et al. (2011). Amplicons were purified with ExoSAP-IT (Thermo Fisher Scientific, Madrid, Spain), and directly sequenced in both directions using primers 8f and 1492r (STABVIDA, Caparica, Portugal). Sequences were assembled and manually corrected using DNASTAR software version 15.3.0.66 (Madison, WI, USA). Isolates were identified to genus/species level by the nearest neighbor in the GenBank “nt” database after alignment with reference 16S rRNA gene sequences using the BLAST algorithm according to Altschul et al. (1997).

### Culture-Independent Characterization of Xylem Bacteria

Aliquots of xylem sap samples (0.5 ml) obtained from macerated xylem chips were placed in PowerBead tubes (DNeasy PowerSoil Kit, QIAGEN) and homogenized 7 min at 50 pulses s^-1^ with the Tissuelyser LT (QIAGEN). Sap extracts were incubated in the lysis buffer for 1 h at 60°C to increase cell lysis, and then processed following the DNeasy PowerSoil Kit manufacturer's instructions.

Aliquots (8 ml) of xylem sap samples extracted with the Scholander chamber were filtered through a 0.22-µm pore MF-Millipore™ filter (Merck Millipore, Madrid, Spain). Then, filters were placed into 1.5-ml Eppendorf tubes and the filtrate resuspended by vortexing for 5 min in the DNeasy PowerSoil Kit lysis buffer, incubated 1 h at 60°C and processed as described before. DNA obtained was quantified and outsourced to the Integrated Microbiome Resource (IMR) at Dalhousie University (Canada) to perform V5-V6 amplicon library sequencing with primers 799F (5'-AACMGGATTAGATACCCKG-3') and 1115R (5'-AGGGTTGCGCTCGTTG-3') and paired-end sequenced by using Illumina MiSeq sequencing platform (V3; PE 2x 300 bp). The ZymoBIOMICS microbial standard (Zymo Research Corp., Irvine, CA, USA) and water (no template DNA) were used as internal positive and negative controls, respectively, for library construction and sequencing. Raw sequence data were deposited in the Sequence Read Archive (SRA) database at the NCBI under BioProject accession number PRJNA574439.

### Statistical and Bioinformatics Analysis

To determine the effects of the olive genotype, type of xylem sap extraction procedure, and culture media, data of culturable bacterial populations obtained in the culture-depended approach were subjected to analysis of variance (ANOVA) using the GLM (General Linear Models) procedure in Statistical Analysis System v. 9.4 (SAS Institute Inc.). Data of culturable bacterial population were log-transformed to fulfill ANOVA assumption. The experiment had a completely randomized design, with olive genotype, type of xylem sap, and culture media as factors with three replications (plates) per experimental unit. Data fulﬁlled the assumptions for ANOVA according to proper statistics. Orthogonal single-degree-of-freedom contrasts were computed to test the effect of selected experimental treatment combinations.

The 16S rRNA sequences obtained were analyzed using the Quantitative Insights into Microbial Ecology bioinformatics pipeline, QIIME2 (version 2018.11; https://view.qiime2.org/) (Caporaso et al., 2010; Bolyen et al., 2018) with default parameters unless otherwise noted. DADA2 pipeline was used for denoising raw fastq paired-end sequences and filtering chimeras. Operational taxonomic units (OTUs) were obtained at 1% of dissimilarity and were taxonomically classified using RDP Bayesian classifier (Wang et al., 2007) against Silva SSU v.132 reference database. Singletons were discarded for taxonomy and statistical analyses.

Differences among bacterial communities derived from the culture-independent approach were calculated in QIIME2 using rarefaction curves of alpha-diversity indexes (including Shannon, Simpson, Faith_PD, and Richness) at the genus level. Alpha and beta diversity as well as alpha rarefaction curves were conducted rarefying all samples to the minimum number of reads found. The Kruskal-Wallis test (*P* <0.05) with FDR correction (Benjamini and Hochberg, 1995) was used to find differences in alpha diversity indexes among the studied factors. Venn diagrams were generated using the “Venn diagram” online tool (http://bioinformatics.psb.ugent.be/webtools/Venn/) and were used to identify shared (core microbiome) or unique taxa according to the type of xylem sap extract and olive genotypes studied, and to compare the culture-dependent and culture-independent approaches. We filtered bacterial taxa and retained those occurring in at least 50% of the samples in a given category. A heat tree summarizing main results was created using Metacoder package in R software (Foster et al., 2017). We unified the taxonomic affiliation derived from BLAST analysis (culture-dependent approach) and Silva SSU v.132 reference database (culture-independent approach) using the NCBI Taxonomy Browser (https://www.ncbi.nlm.nih.gov/Taxonomy/Browser/wwwtax.cgi?mode=Root). Taxonomic abundances within each identified Phylum to genus level were visualized using Krona hierarchical data browser (Ondov et al., 2011).

A non-supervised principal component analysis (PCA) and multivariate hierarchical clustering analysis (using Pearson's correlation to measure distance and the Ward clustering algorithm) were performed using the OTU frequency matrixes at the genus level derived from the culture-dependent and culture-independent approach with the online tool MetaboAnalyst 4.0 (http://www.metaboanalyst.ca; Chong et al., 2018).

## Results

### Bacterial Abundance and Alpha Diversity Measures

In the culture-dependent approach, bacterial population densities in xylem sap extracted from woody chips ranged from 40 to 1,920 colony forming units (cfu)/ml, whereas those obtained from xylem sap samples obtained with the Scholander pressure chamber ranged from 10 to 610 cfu/ml. Only two factors in the study were found to be significant [olive genotype (*F*=12.6, *P* < 0.0001) and type of xylem sap extraction method (*F =* 22.4, *P* < 0.0001), representing 16.6% and 29.7% of the mean square error (MSE) in the model, respectively]. Only the interaction type of xylem sap extraction method by olive genotype was significant (*F =* 35.67, *P* < 0.0001) representing 47.2% of the MSE in the model.

**Figure 1:**
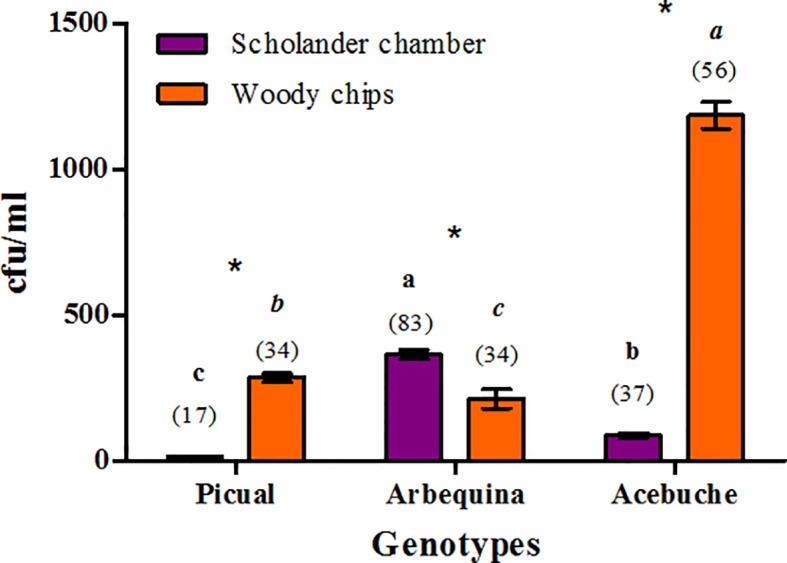
Culturable bacterial population densities [colony forming units (cfu)/ml] present in xylem sap of different olive genotypes extracted with the Scholander chamber or from woody chips macerates from three independent trees per genotype, irrespective of the culture medium used. The number of bacterial isolates selected for 16S taxonomic identification for each treatment is indicated between brackets. For each extraction method, bars with the same letter do not differ significantly among olive genotypes. The “*” indicate for each genotype the existence of significant differences at *P≤* 0.05 between both xylem sap extraction methods.

A significantly higher (*F* > 32.09, *P* < 0.0001) bacterial population density was estimated on xylem samples extracted from woody chips compared to xylem sap extracted with the Scholander chamber for “Acebuche” and “Picual,” while the opposite occurred for “Arbequina” (*F* = 17.13, *P* = 0.0001) for both xylem extraction procedures (**Figure 1**). “Arbequina” showed a significantly higher (*F* > 10.45, *P* < 0.0021) bacterial population than “Picual” and “Acebuche” when xylem sap was obtained with the Scholander chamber, whereas “Acebuche” showed a significantly higher (*F* > 9.24, *P* < 0.0037) bacterial population than “Arbequina” and “Picual” when the xylem sap was extracted from woody chips macerates (**Figure 1**).

Maximal recoveries of bacterial population were observed with R2A medium (478.9 ± 131.9 cfu/ml), and decreased with BCYE (352.2 ± 101.3 cfu/ml), PD2 (335.0 ± 97.6 cfu/ml), and NA (270.0 ± 82.0 cfu/ml), in that order, although no significant differences were found among culture media (*F =* 1.98, *P =* 0.1298) (*data not shown*).

A total of 261 bacterial isolated were selected for further study from xylem sap and xylem chips extracts after cultivation in NA, PD2, R2A, or BCYE culture media (**Figure 1**; **Table S1**). Out of those, 137 bacterial isolates were from xylem sap extracted with the Scholander chamber while 124 were selected from xylem chips extracts. From the 261 isolates, 51 and 117 bacterial isolates were recovered from cultivated olives “Picual” and “Arbequina,” respectively, and 93 bacterial isolates from the wild olive genotype “Acebuche” (**Figure 1**).

In the culture-independent approach, Illumina MiSeq sequencing analysis resulted in a total of 21,411 good quality reads with an average of 334 bp after removal of chimeras, unassigned or mitochondrial reads. No chloroplasts reads were detected in our samples. A total of 58 OTUs were identified for all treatments, out of which 48 OTUs were retained after rarefying all data to 1,234 sequences (the minimum number of reads obtained in one of the samples) and singleton removal.

Rarefaction curves of observed OTUs (Richness) displayed significantly higher (*H* = 12.79; *P* < 0.0003) values in xylem sap extracted with the Scholander chamber as compared to the woody chips maceration method. Interestingly, although “Picual” was the olive cultivar showing the lowest number of culturable bacteria (**Figure 1**), it presented a significantly higher number of OTUs for both extraction methods (*H =* 20.19, *P* < 0.0001), followed by “Acebuche” and “Arbequina” (**Figure S1**). Maximum values of Good's coverage of 1.0 were obtained for all samples (*data not shown*).

Alpha diversity indices (Richness, Shannon, Faith_PD and Simpson) did not show global significant differences between xylem sap extraction methods (*H* < 3.185, *P* > 0.074), among olive genotypes (*H* < 1.977, *P* > 0.372) or their interaction (*H* < 6.591, *P* > 0.253) (*data not shown*).

### Composition of Xylem Sap Bacterial Communities

**Figure 2:**
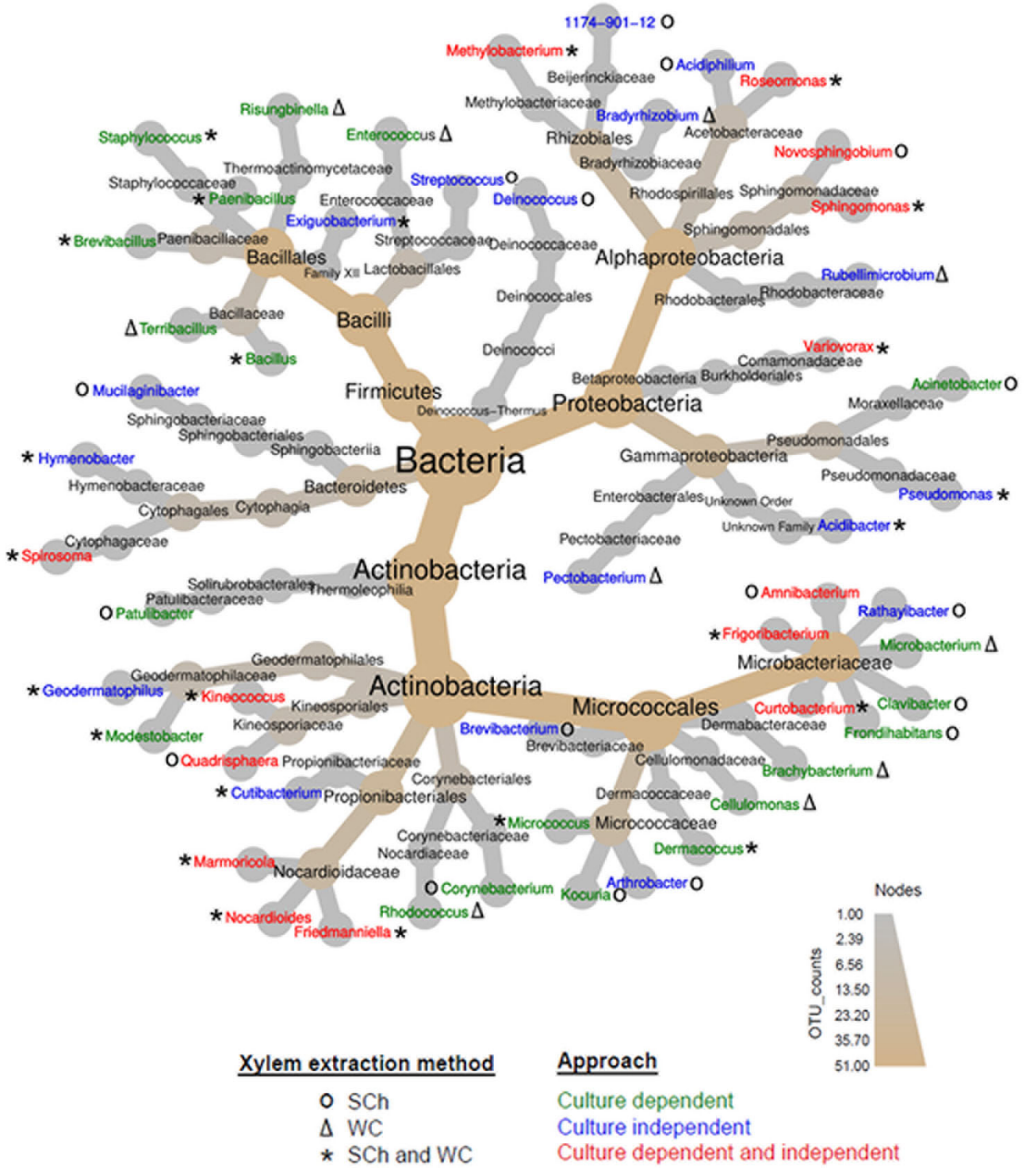
Heat tree of the abundance of bacterial taxa at different ranks present in olive xylem sap and determined using culture-dependent and culture-independent approaches and extracted with the Scholander chamber (SCh) or from woody chips (WC) macerates. The size and color of nodes and edges are correlated with the abundance of taxa. The central nodes are the total of all the other nodes in the tree for each phylum.

In the culture-dependent approach, a total of 4 phyla, 7 classes, 14 orders, 22 families, and 34 genera were identified (**Figure 2**). Significant differences (*P* < 0.05) were found in the number of genera isolated according to the culture media (**Figure S2**). Out of the total of 34 bacterial genera identified, 11 genera were detected in AN, 17 in BCYE and PD2, and 24 in R2A. Seven of the genera (*Bacillus, Brachybacterium, Curtobacterium, Dermacoccus, Frigoribacterium, Methylobacterium*, and *Sphingomonas*) were isolated in all culture media, whereas seven, four and four, and one genera were unique to R2A, PD2 and AN, and BCYE, respectively. Those unique genera corresponded normally to single isolates (**Figure S2**; **Table S1**).

**Figure 3:**
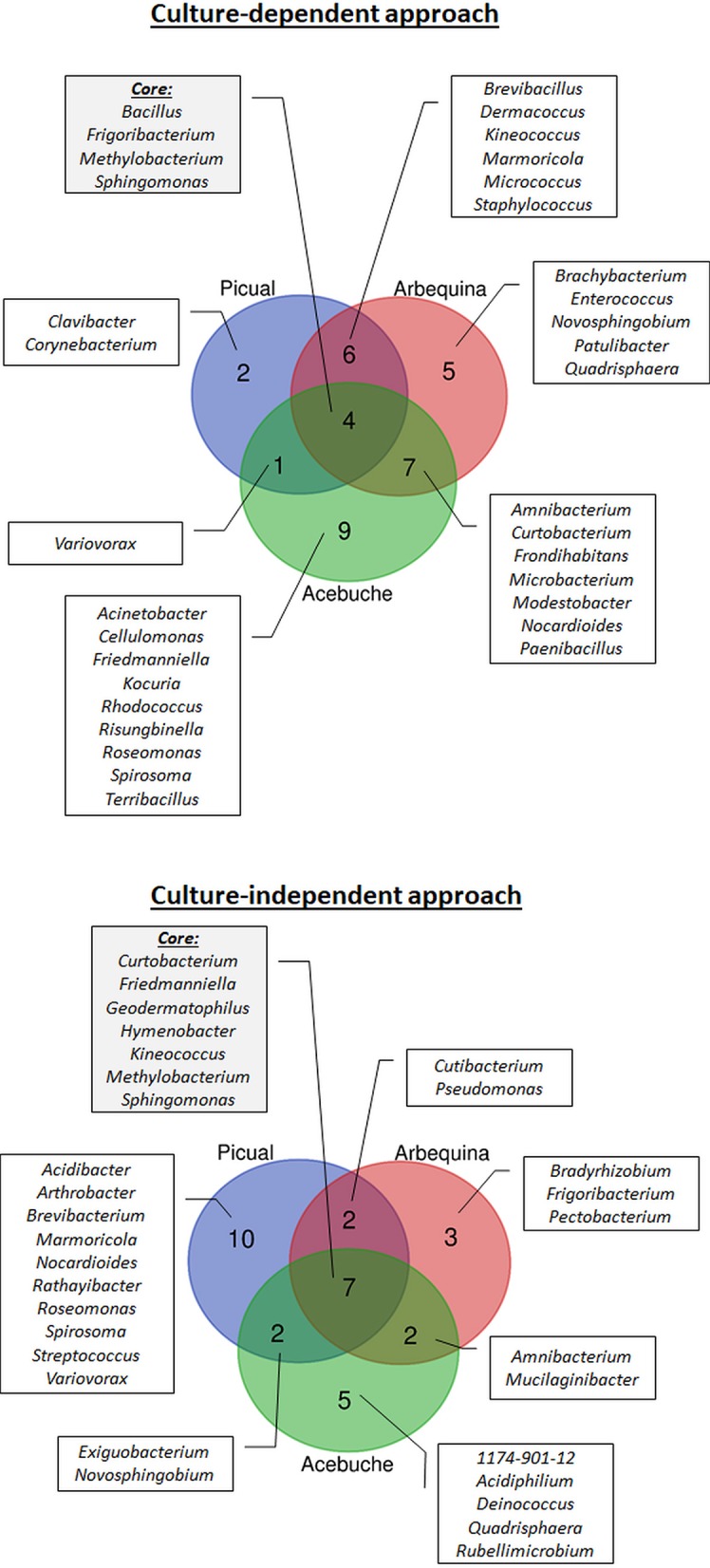
Prevalence Venn diagram showing the unique and shared bacterial genera obtained using culture-dependent approaches (upper panel) or culture-independent approach (lower panel) in olive xylem sap samples when compared by olive genotype (“Acebuche,” “Arbequina,” and “Picual”).

**Figure 4:**
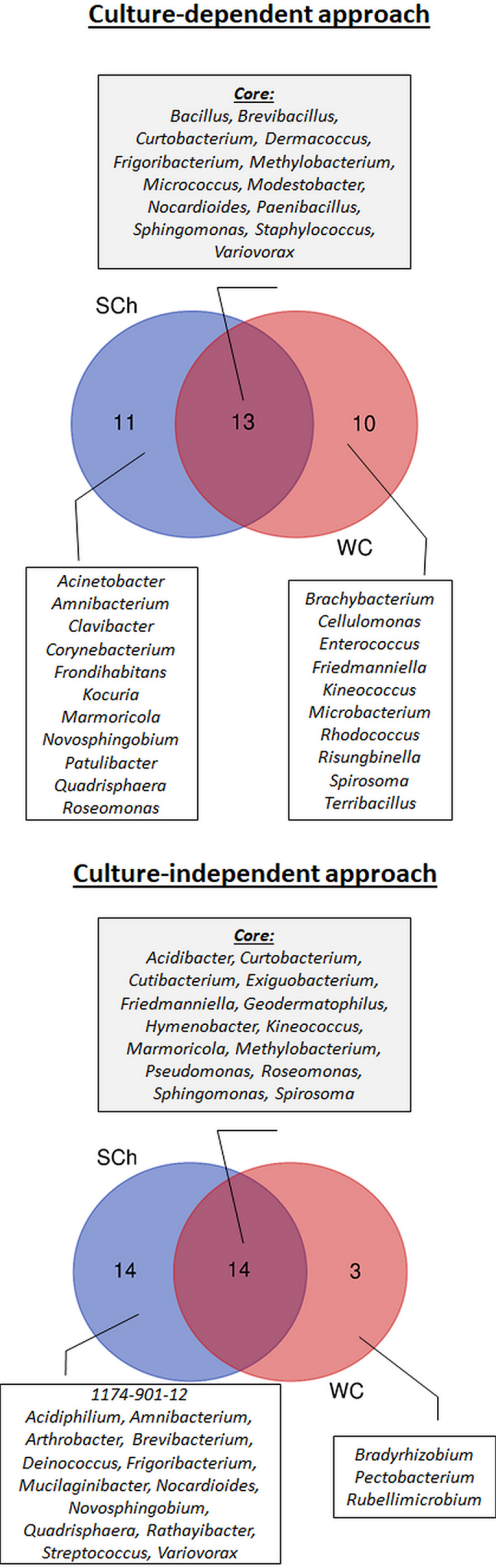
Prevalence Venn diagram showing the unique and shared bacterial genera obtained using culture-dependent approaches (upper panel) or culture-independent approach (lower panel) in olive xylem sap samples when compared by xylem sap extraction method [Scholander chamber (SCh) or wood chips (WC) maceration].

Regarding olive cultivars, 13 genera were identified in “Picual,” 22 in “Arbequina,” and 21 in “Acebuche.” Only four bacterial genera (11.8% of the total identified) were shared among the three olive genotypes and were present in more than 50% of all samples (Core bacterial genera: *Bacillus, Frigoribacterium, Methylobacterium, Sphingomonas*), six were shared between the two cultivated olive genotypes (*Brevibacillus, Dermacoccus, Kineococcus, Marmoricola, Micrococcus, Staphylococcus*), seven were shared between the wild olive genotype “Acebuche” and “Arbequina” (*Amnibacterium, Curtobacterium, Frondihabitans, Microbacterium, Modestobacter, Nocardioides, Paenibacillus*), and only one (*Variovorax*) between “Acebuche” and “Picual” (**Figure 3**). “Acebuche” showed the highest number of unique genera (nine in total). Finally, regarding xylem sap extraction method, 13 (38.2%) of the culturable bacterial genera (*Bacillus, Brevibacillus, Curtobacterium, Dermacoccus, Frigoribacterium, Methylobacterium, Micrococcus, Modestobacter, Nocardioides, Paenibacillus, Sphingomonas, Staphylococcus* and *Variovorax*) were isolated by both extraction methods, whereas 11 (32.4%) and 10 (29.4%) of bacterial genera were unique to the Scholander chamber extraction method (*Acinetobacter, Amnibacterium, Clavibacter, Corynebacterium, Frondihabitans, Kocuria, Marmoricola, Novosphingobium, Patulibacter, Quadrisphaera, Roseomonas*) or the woody chips extraction method (*Brachybacterium, Cellulomonas, Enterococcus, Friedmanniella, Kineococcus, Microbacterium, Rhodococcus, Risungbinella, Spirosoma, Terribacillus*), respectively (**Figure 4**).

When using the culture-independent approach, a total of 5 phyla, 8 classes, 17 orders, 23 families, and 31 genera were identified (**Figure 2**). Twenty-one of the genera were identified in “Picual,” 14 in “Arbequina,” and 16 in “Acebuche” (**Figure 3**). Only seven bacterial genera (22.6% of the total identified) were shared among the three olive genotypes (Core bacterial genera: *Curtobacterium, Friedmanniella, Geodermatophilus, Hymenobacter, Kineococcus, Methylobacterium, Sphingomonas*), two were shared between the two cultivated olive genotypes (*Cutibacterium* and *Pseudomonas*), two were shared between the wild olive genotype “Acebuche” and “Arbequina” (*Amnibacterium* and *Mucilaginibacter*), and two (*Exiguobacterium* and *Novosphingobium*) between “Acebuche” and “Picual.” “Picual” showed the highest number of unique genera (10 in total) (**Figure 3**). Regarding xylem sap extraction method 14 (45.2%) of the bacterial genera identified were isolated by both extraction methods (*Acidibacter, Curtobacterium, Cutibacterium, Exiguobacterium, Friedmanniella, Geodermatophilus, Hymenobacter, Kineococcus, Marmoricola, Methylobacterium, Pseudomonas, Roseomonas, Sphingomonas, Spirosoma*), whereas 14 (45.2%) and 3 (0.10%) of bacterial genera were unique to the Scholander chamber extraction method (*1174-901-12, Acidiphilium, Amnibacterium, Arthrobacter, Brevibacterium, Deinococcus, Frigoribacterium, Mucilaginibacter, Nocardioides, Novosphingobium, Quadrisphaera, Rathayibacter, Streptococcus, Variovorax*) or the woody chips extraction method (*Bradyrhizobium, Pectobacterium, Rubellimicrobium*), respectively (**Figure 4**).

Four of the phyla, *Actinobacteria*, *Bacteroidetes*, *Firmicutes*, and *Proteobacteria*, were obtained both by the culture-dependent and culture-independent approaches. Only the phylum *Deinococcus-Thermus* emerged exclusively when using the culture-independent approach (**Figure 2**). At the Class level *Actinobacteria*, *Bacilli*, *Alphaproteobacteria Cytophagia, Betaproteobacteria*, and *Gammaproteobacteria* were identified following both approaches. *Thermophilia* class was detected only in the culture-dependent approach while *Sphingobacteriia* and *Deinococci* classes were identified only when using the NGS procedure (**Figure 2**). At the genus level, a total of 51 genera were identified combining both culture-dependent and culture-independent approaches; 20 and 17 genera were exclusive of the culture-dependent and culture-independent approaches, respectively, and 14 bacterial genera were shared by both methodologies (**Figure 2**).

### Bacterial Abundance Distribution

*Actinobacteria* phylum presented the highest relative abundance in the culture-dependent approach (46.37%) followed by *Proteobacteria* (34.09%), *Firmicutes* (19.15%), and *Bacteroidetes* (0.38%) (**Figure S3**). Differently, *Proteobacteria* showed more than half of the total bacterial genera in the culture-independent approach (56.25%), followed by *Bacteroidetes* (24.76%), *Actinobacteria* (13.44%), *Firmicutes* (0.77%), and *Deinococcus-Thermus* (0.21%) (**Figure S4**). At the family level, the most abundant bacterial families were *Microbacteriaceae* (34.48%), *Methylobacteriaceae* (16.86%), *Sphingomonadaceae* (31.44%), and *Bacillaceae* (13.03%) when using the culture-dependent approach, whereas *Sphingomonadaceae* (31.44%), *Hymenobacteraceae* (24.11%), and *Methylobacteriaceae* (22.36%) were the most abundant when using the culture-independent approach. Focusing on the culture-dependent methodology, the most abundant genera were *Frigoribacterium* (18.77%), *Methylobacterium* (16.36%), *Sphingomonas* (14.56%), *Bacillus* (12.64%), and *Curtobacterium* (11.88%), while when we used the NGS approach, the most abundant genera identified were *Sphingomonas* (30.10%), *Hymenobacter* (24.11%), and *Methylobacterium* (22.36%) (**Figures S3** and **S4**).

### Bacterial Community Structure

**Figure 5:**
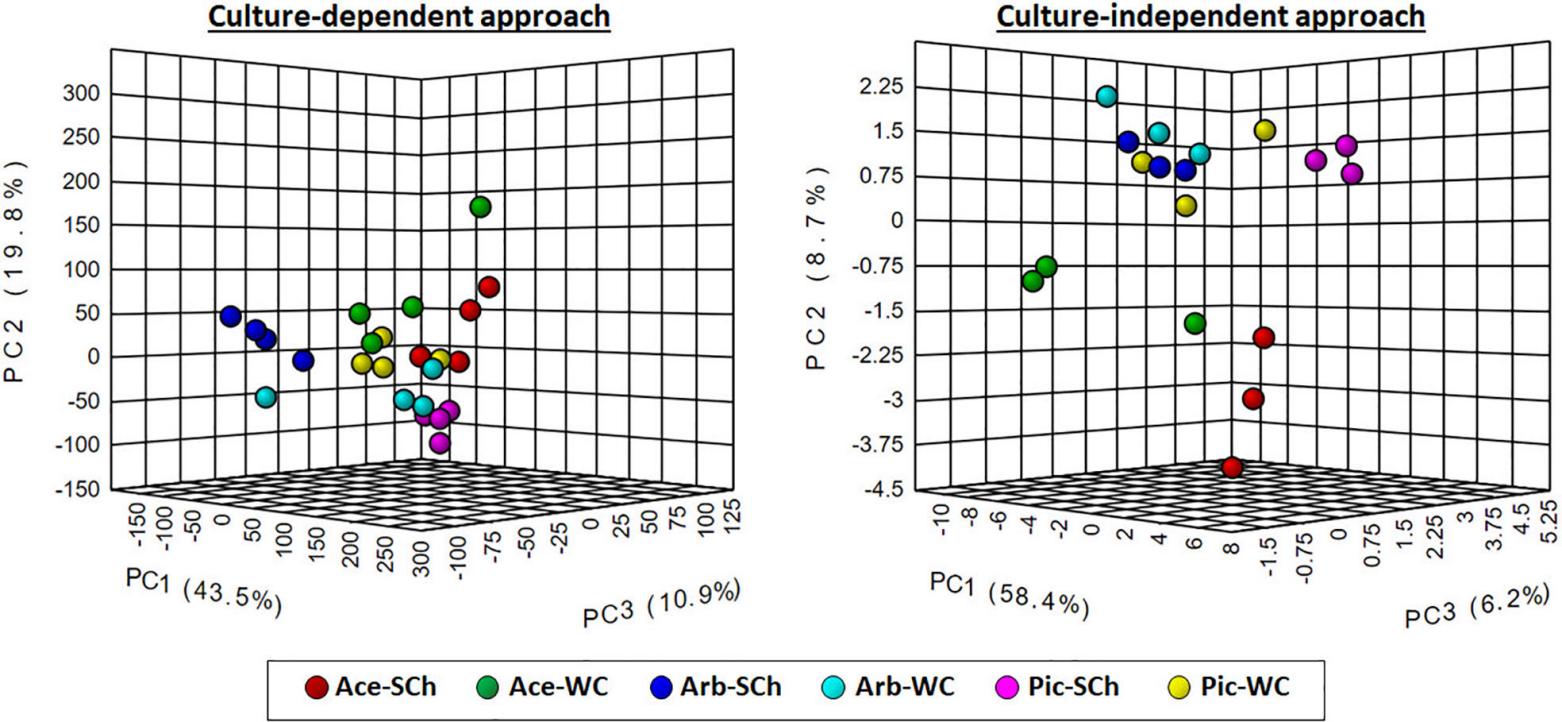
Principal component analysis of the relative abundance of bacterial genera obtained using culture-dependent approaches (left panel) or culture-independent approach (right panel) in olive xylem sap samples when compared by olive genotype (Ace: “Acebuche,” Arb: “Arbequina,” and Pi: “Picual”) or by xylem sap extraction method [Scholander chamber (SCh) or wood chips (WC) maceration].

Hierarchical clustering analysis and PCA using OTU frequencies at the genus level differentiated xylem bacterial communities according to the sap extraction method or the olive genotype irrespective of the culture approach used (**Figure 5**; **Figure S5**). However, these differences were more noticeable when using the culture-independent approach, for which there was a clear trend to group the bacterial communities first by olive genotype and then by extraction method with only one exception, and with “Acebuche” showing the most distant bacterial communities as compared to the cultivated olive genotypes.

## Discussion

This study resulted in an initial characterization of the bacterial microbiome of the xylem sap from two cultivated and one wild olive genotypes following culture-dependent and -independent approaches, thus providing new insights into the olive microbiome profile, especially in a vascular system where some of the most destructive olive pathogens thrive. Our results suggest that the characterization of the xylem bacterial endophyte community composition is strongly dependent on the use of culture-dependent or -independent approaches and on the xylem sap extraction method used (Scholander chamber vs. woody chips). This initial characterization of olive xylem bacterial microbiome represents a first step making inroads into a potentially promising strategy for the identification of prospective biological control agents well adapted to the ecological niche in which these organisms would be potentially applied.

We found that the population of culturable xylem-associated bacteria ranged from 10^1^ to 2×10^3^ cfu/ml, depending on the olive genotype and the extraction method. These population ranges are in agreement with those obtained for other woody crops such as citrus and grapes (Bell et al., 1995; Gardner et al., 1982). Although the estimates obtained per gram of homogenized vessel xylem tissue are not strictly comparable to data obtained for the vacuum Scholander chamber, mainly due to differences in the amount of plant tissue sampled, the population levels obtained when using the former method appeared to be much higher in general, even when the amount of tissue sampled was smaller. Microscopic analysis of xylem samples from grapes and citrus (Bell et al., 1995; Gardner et al., 1982) revealed that many bacteria remain attached to xylem vessel walls with fibrillar material. These observations might also explain our differences in olive, not only quantitatively but also qualitatively, i.e., the differences noticed in bacterial genera retrieved by both extraction methods. It may be possible that certain groups of xylem-inhabiting bacteria show, or certain environmental conditions induce, a preference for planktonic growth as compared to a static-biofilm associated growth on xylem tissue. Since biofilm formation in plants, and specifically on xylem tissues, is associated with both symbiotic (beneficial) and pathogenic responses, these potential differences in growth styles or changes due to environmental conditions within the xylem vessels should be taken into consideration in future studies (Cruz et al., 2012; Bogino et al., 2013).

Olive is one of the most ancient domesticated plants, widely distributed in Roman times and its culture documented since ca. 6,000 years ago (Lavee, 1990). A key feature of cultivated plants is represented by the process of domestication and breeding, with a net reduction of genetic diversity and the promotion of growth with external inputs, which may interfere with the establishment of microbiome assemblages (Doebley et al., 2006). We found differences in the microbiome community composition associated to xylem-sap depending of the olive genotype, indicating a more different bacterial composition of wild olive as compared to both cultivated olive genotypes. Several studies with domesticated crops have indicated differences in microbiome community composition between wild accessions and modern cultivated varieties, mainly studied in herbaceous species (e.g., barley, bean maize, and rice; Peiffer et al., 2013; Bulgarelli et al., 2015; Edwards et al., 2015; Pérez-Jaramillo et al., 2017), less so in woody crops (e.g., Bullington and Larkin, 2015; Deyett et al., 2017; Yang et al., 2017), with only a few studies in olive (Aranda et al., 2011; Montes-Borrego et al., 2013; Montes-Borrego et al., 2014; Müller et al., 2015). Although this study shows some evidence on the existence of significant differences in population densities and community composition of the xylem-limited microbiome according to the olive genotype, this observation should be further explored including a higher number of olive cultivars growing under different environmental conditions.

Our NGS results with olive xylem indicated the presence of endophytic bacteria from the phyla *Actinobacteria*, *Firmicutes*, *Bacteroidetes*, *Proteobacteria*, and *Deinococcus-Thermus*. However, while *Actinobacteria* and *Proteobacteria* bacteria were present in all three olive genotypes, only “Picual” contained *Firmicutes* and *Bacteroidetes*, and only “Acebuche” contained bacteria from the *Deinococcus-Thermus* phylum. These results are in accordance with those obtained previously following similar approaches from olive leaves and xylem sap (Müller et al., 2015; Fausto et al., 2018; Sofo et al., 2019) as well as from other woody plants, like grapes, oak, poplar, and *Pinus* (Uroz et al., 2010; Bonito et al., 2014; Cregger et al., 2018; Deyett and Rolshausen, 2019). Interestingly, our results were more in agreement with those obtained by Deyett and Rolshausen (2019) that found similar proportions for Proteobacteria and Actinobacteria on grapevine xylem sap than those on olives obtained by Fausto et al. (2018), when using a similar xylem sap extraction method with a Scholander pressure chamber. Some differences were also found among those studies at the genus level. Thus, Deyett and Rolshausen (2019) found bacteria in the Enterobacteriaceae family, and belonging to *Streptococcus, Bacteroides*, *Bacillus*, *Acinetobacter*, and *Pseudomonas* genera as the most abundant on grapewine sap, whereas Fausto et al. (2018) identified some bacterial genera in the olive sap common to our study (e.g., *Curtobacterium, Hymenobacter, Methylobacterium,* and *Sphingomonas*), although they did not provide their relative abundance. Those differences may be due to the fact that different primers targeting different variable regions of the 16 rRNA were used for amplification in each study, or to the differences on environmental growing conditions and the plant genotypes evaluated.

In our study the bacterial genera detected in the highest proportions were, in this order, *Sphingomonas, Hymenobacter*, and *Methylobacterium*. Interestingly, despite the abundance of *Hymenobacter* determined by NGS, we did not isolate it in culture, although this bacterium has been readily isolated from stem and leaves of other plant species (Izhaki et al., 2013; Ding and Melcher, 2016; Durand et al., 2018; Ginnan et al., 2018). Other abundant genera identified through NGS were *Kineococcus* and *Friedmanniella*, both in xylem sap and woody chips. However, they were only isolated in culture from woody chips, which might indicate the presence of different species of cultivable or non-cultivable bacteria of *Kineococcus* and *Friedmanniella* in the olive xylem microbiota or a preference of these genera for a stationary growth within the xylem. Cultivated members of these genera had been previously isolated from plant stems (Qin et al., 2009; Tuo et al., 2016) and, as members of the *Actinobacteria* phylum, they have been proposed to increase agricultural productivity through plant-growth promotion and to have the potential to be used as an alternative to chemical fertilizers (Palaniyandi et al., 2013; Hamedi and Mohammadipanah, 2015).

Traditional approaches for studying the diversity of plant microbial communities have relied on cultured-dependent approximations. Although the advances in -omic and NGS technologies over the last decade have enabled the culture-independent study of plant microbiomes, we believe that both approaches should be used in parallel to provide deeper knowledge and exploitation of the plant-associated microbiome, especially for perennial crops (Gagliardi et al., 2001; Aranda et al., 2011; Mendes et al., 2011; Jackson et al., 2013; Dissanayake et al., 2018). With olive, our results using the culture-dependent approach indicated that endophytic bacteria from phyla *Actinobacteria*, *Firmicutes*, *Bacteroidetes*, and *Proteobacteria* can be readily isolated from the different plant genotypes. Considering the unique microbial communities found for each olive genotype, however, no *Bacteroidetes* could be isolated from “Arbequina,” while for “Picual,” unique bacteria belonging to *Actinobacteria* phylum were isolated. These results are in line with the gross taxonomic bacterial distribution of isolates already identified in the olive rhizosphere and phyllosphere (Ercolani, 1991; Aranda et al., 2011), as well as with the composition of bacterial endophytes identified in other woody plants, such as citrus, grapevine, or poplar (Bell et al., 1995; Compant et al., 2011; Azevedo et al., 2016; Durand et al., 2018). At the genus level, *Sphingomonas*, *Methylobacterium*, *Curtobacterium*, *Frigoribacterium*, and *Bacillus* were among the most abundant culturable genera. *Frigoribacterium* are endophytic bacteria isolated from poplar trees (Ulrich et al., 2008) and from potato, where they have an antagonistic capacity against phytopathogenic fungi (Berg and Hallmann, 2006). *Bacillus* bacteria are well-known rhizosphere and endosphere colonizers with diverse antagonistic activities against plant pathogens (Kumar et al., 2012). The widespread occurrence of the *Sphingomonas* and *Methylobacterium* genera has been described as crucial in plants due to a diverse range of functions, including degradation of certain contaminants and facilitation of soil nutrient cycling and plant growth (White et al., 1996; Chen et al., 2016). This may explain their ample distribution in the plant kingdom, from legume species (Khan et al., 2014) to woody plants (Van Aken et al., 2004) and throughout different plant organs, such as seed, leaf, and flower tissues (Kim et al., 1998). Interestingly, *Methylobacterium* isolates have been shown to exhibit antagonistic properties that induce resistance against the attack of diverse fungi (Rajendran et al., 2009; Ardanov et al., 2012) or that, more importantly, can modify the response of the xylem-inhabiting bacterial pathogen *X. fastidiosa* in citrus, resulting in a reduction of Citrus Variegated Chlorosis (CVC) symptoms (Azevedo et al., 2016). Similarly, *Curtobacterium* has been also described in citrus plants as an endophyte interacting with *X. fastidiosa* and reducing the severity of the CVC symptoms (Lacava et al., 2007). All these characteristics make several of the bacterial isolates from xylem sap of olive obtained in this study good candidates for testing their potential to confer protection against the xylem-inhabiting pathogens *X. fastidiosa* and *V. dahliae* in future research.

Although several studies on woody plant species have revealed the power of using culture-independent approaches for revealing previously unappreciated microbial diversity on xylem samples (Müller et al., 2015; Deyett et al., 2017; Fausto et al., 2018; Deyett and Rolshausen, 2019; Sofo et al., 2019), our study is unique in that culture-dependent and -independent methods were compared directly and simultaneously (i.e., applied to the same samples) in different genotypes of the host plant using two different extraction methods, to perform a quantitative and qualitative assessment of the xylem bacterial microbiome. Although knowing the specific limitations of each of the culture-dependent and culture-independent approaches, and the short number of olive genotypes and samples evaluated, our study is relevant in that it paves the way for future studies on the identification and characterization of olive xylem sap endophytic bacteria that could be involved in plant defense by acting as biocontrol agents against diverse xylem-inhabiting pathogens and by promoting plant health and growth promotion of olive plants. This information, in turn, will be generally useful for innovative microbiome research based both on culture-dependent and culture-independent approaches wherever vascular plant pathogens and nutrient requirements are involved.

## Data Availability Statement

The raw sequence data have been deposited in the Sequence Read Archive (SRA) database at the NCBI under BioProject accession number PRJNA574439.

## Author Contributions

MA-M and BL: conceived research, performed statistical and bioinformatics analyses, interpreted results, and wrote the manuscript. CO-G, MA-M, and CH: prepared materials and equipment and performed the experiments. JN-C and JI: contributed to reviewing the manuscript and interpreting results. All authors viewed and approved the manuscript.

## Funding

This study was funded by project AGL2016-75606-R (Programa Estatal de I+D Orientado a los Retos de la Sociedad from the Spanish Government and the Spanish State Research Agency and FEDER-EU) and Project XF-ACTORS (grant 727987) from the European Union's Horizon 2020 Framework Research Programme. MA-M is a recipient of a research fellowship BES-2017-082361 from the Spanish Ministry of Economy and Competitiveness.

## Conflict of Interest

The authors declare that the research was conducted in the absence of any commercial or financial relationships that could be construed as a potential conflict of interest.

## Supplementary Materials

The Supplementary Material for this article can be found online at: https://www.frontiersin.org/articles/10.3389/fpls.2019.01708/full#supplementary-material

Figure S1 Comparison of rarefaction curves of observed OTUs present in the xylem sap of ‘Picual’ (Pic), ‘Arbequina’ (Arb), and ‘Acebuche’ (Ace) olive genotypes extracted with the Scholander chamber (SCh) or from woody chips macerates (WC). Error bars represent standard derivation of three independent tree replicates. Data were rarified to 1,234 sequences.

Figure S2 Prevalence Venn diagram and table of cases showing the unique and shared bacterial genera obtained using culture-dependent approaches when compared by culture isolation media.

Figure S3 Bacterial taxonomic abundances (%) from phylum to genus level derived from culture-dependent approach.

Figure S4 Bacterial taxonomic abundances (%) from phylum to genus level derived from culture-independent approach.

Figure S5 Hierarchical cluster analysis and heat-map of the relative abundance of bacterial genera obtained using culture-dependent approaches (left panel) or culture-independent approach (right panel) in olive xylem sap samples when compared by olive genotype (Ace: ‘Acebuche’, Arb: ‘Arbequina’ and Pi: ‘Picual’) or by xylem sap extraction method (Scholander chamber (SCh) or wood chips (WC) maceration). The relative abundance of the bacteria are scaled by a color gradient bar.

Table S1 Bacterial isolates obtained from xylem sap of olive in the present study, indicating the sap extraction method, culture media of isolation and their identification at genus level based on partial sequencing of 16S rRNA and BLAST analysis.

## References

[B1] Alexou M. Peuke A. (2013). Methods for xylem sap collection. Methods Mol. Biol. 953, 195–207. 10.1007/978-6561-62703-152-3_13 23073885

[B2] Almeida R. P. P. De La Fuente L. Koebnik R. Lopes J. R. S. Parnell S. Scherm H. (2019). Addressing the New Global Threat of Xylella fastidiosa. Phytopathology 109, 172–174. 10.1094/PHYTO-12-18-0488-FI 30721121

[B3] Altschul S. F. Madden T. L. Schäffer A. A. Zhang J. Zhang Z. Miller W. (1997). Gapped BLAST and PSI-BLAST: a new generation of protein database search programs. Nucleic Acids Res. 25, 3389–3402. 10.1093/nar/25.17.3389 9254694 PMC146917

[B4] Aranda S. Montes-Borrego M. Jiménez-Díaz R. M. Landa B. B. (2011). Microbial communities associated with the root system of wild olives (*Olea europaea* L. subsp. *europaea* var. *sylvestris*) are good reservoirs of bacteria with antagonistic potential against Verticillium dahliae. Plant Soil 343, 329–345. 10.1007/s11104-011-0721-2

[B5] Ardanov P. Sessitsch A. Häggman H. Kozyrovska N. Pirttilä A. M. (2012). Methylobacterium-Induced Endophyte Community Changes Correspond with Protection of Plants against Pathogen Attack. PloS One 7, e46802. 10.1371/journal.pone.0046802 23056459 PMC3463518

[B6] Azevedo J. Araújo W. L. Lacava P. T. (2016). The diversity of citrus endophytic bacteria and their interactions with Xylella fastidiosa and host plants. Genet. Mol. Biol. 39, 476–491. 10.1590/1678-4685-GMB-2016-0056. 27727362 PMC5127157

[B7] Bell C. R. Dickie G. A. Harvey W. L. G. Chan J. W. Y. F. (1995). Endophytic bacteria in grapevine. Can. J. Microbiol. 41, 46–53. 10.1139/m95-006

[B8] Benjamini Y. Hochberg Y. (1995). Controlling the false discovery rate: a practical and powerful approach to multiple testing. J. R. Stat. Soc Ser. B 57, 289–300. Available at: http://www.jstor.org/stable/2346101.

[B9] Berg G. Hallmann J. (2006). Control of Plant Pathogenic Fungi with Bacterial Endophytes. Eds. Schulz B. J. E. Boyle C. J. C. Sieber T. N. (Berlin, Heidelberg: Springer Berlin Heidelberg), Microb. Root Endophytes 53–69. 10.1007/3-540-33526-9_4

[B10] Berg G. Grube M. Schloter M. Smalla K. (2014). Unraveling the plant microbiome: looking back and future perspectives. Front. Microbiol. 5, 148. 10.3389/fmicb.2014.00148 24926286 PMC4045152

[B11] Bogino C. P. Oliva D. M. Sorroche G. F. Giordano W. (2013). The role of bacterial biofilms and surface components in plant-bacterial associations. Int. J. Mol. Sci. 14, 15838–15859. 10.3390/ijms140815838 23903045 PMC3759889

[B12] Bolyen E. Rideout J. R. Dillon M. R. Bokulich N. A. Abnet C. Al-Ghalith G. A. (2018). QIIME 2: Reproducible, interactive, scalable, and extensible microbiome data science. PeerJ Prepr. 6, e27295v2. 10.7287/peerj.preprints.27295v2 PMC7015180 31341288

[B13] Bonito G. Reynolds H. Robeson M. S. II Nelson J. Hodkinson B. P. Tuskan G. (2014). Plant host and soil origin influence fungal and bacterial assemblages in the roots of woody plants. Mol. Ecol. 23, 3356–3370. 10.1111/mec.12821 24894495

[B14] Bulgarelli D. Garrido-Oter R. Münch P. C. Weiman A. Dröge J. Pan Y. (2015). Structure and function of the bacterial root microbiota in wild and domesticated barley. Cell Host Microbe 17, 392–403. 10.1016/j.chom.2015.01.011 25732064 PMC4362959

[B15] Bullington L. S. Larkin B. G. (2015). Using direct amplification and next-generation sequencing technology to explore foliar endophyte communities in experimentally inoculated western white pines. Fungal Ecol. 17, 170–178. 10.1016/j.funeco.2015.07.005

[B16] Calderon R. Lucena C. Trapero-Casas J. L. Zarco-Tejada P. J. Navas-Cortes J. A. (2014). Soil temperature determines the reaction of olive cultivars to Verticillium dahliae pathotypes. PloS One 9, e110664. 10.1371/journal.pone.0110664 25330093 PMC4201566

[B17] Caliz J. Montes-Borrego M. Triadó-Margarit X. Metsis M. Landa B. B. Casamayor E. O. (2015). Influence of edaphic, climatic, and agronomic factors on the composition and abundance of nitrifying microorganisms in the rhizosphere of commercial olive crops. PloS One 10, e0125787. 10.1371/journal.pone.0125787 25950678 PMC4423868

[B18] Caporaso J. G. Kuczynski J. Stombaugh J. Bittinger K. Bushman F. D. Costello E. K. (2010). QIIME allows analysis of high-throughput community sequencing data. Nat. Methods 7, 335. 10.1038/nmeth.f.303 20383131 PMC3156573

[B19] Chen L. Brookes P. C. Xu J. Zhang J. Zhang C. Zhou X. (2016). Structural and functional differentiation of the root-associated bacterial microbiomes of perennial ryegrass. Soil Biol. Biochem. 98, 1–10. 10.1016/j.soilbio.2016.04.004

[B20] Chong J. Soufan O. Li C. Caraus I. Li S. Bourque G. (2018). MetaboAnalyst 4.0: towards more transparent and integrative metabolomics analysis. Nucleic Acids Res. 46, W486–W494. 10.1093/nar/gky310 29762782 PMC6030889

[B21] Compant S. Mitter B. Colli-Mull J. G. Gangl H. Sessitsch A. (2011). Endophytes of grapevine flowers, berries, and seeds: identification of cultivable bacteria, comparison with other plant parts, and visualization of niches of colonization. Microb. Ecol. 62, 188–197. 10.1007/s00248-011-9883-y 21625971

[B22] Cregger M. A. Veach A. M. Yang Z. K. Crouch M. J. Vilgalys R. Tuskan G. A. (2018). The populus holobiont: dissecting the effects of plant niches and genotype on the microbiome. Microbiome 6, 1–14. 10.1186/s40168-018-0413-8 29433554 PMC5810025

[B23] Cruz L. F. Cobine P. A. De La Fuente L. (2012). Calcium increases xylella fastidiosa surface attachment, biofilm formation, and twitching motility. Appl. Environ. Microbiol. 78, 1321–1331. 10.1128/AEM.06501-11 22194297 PMC3294462

[B24] Davis M. J. French W. J. Schaad N. W. (1981). Axenic culture of the bacteria associated with phony disease of peach and plum leaf scald. Curr. Microbiol. 6, 309–314. 10.1007/BF01566883

[B25] Deyett E. Roper M. C. Ruegger P. Yang J.-I. Borneman J. Rolshausen P. E. (2017). Microbial landscape of the grapevine endosphere in the context of pierce's Disease. Phytobiomes J. 1, 138–149. 10.1094/PBIOMES-08-17-0033-R

[B67] Deyett E. Rolshausen P. E. (2019). Temporal dynamics of the sap microbiome of grapevine under high pierce's disease pressure. Front. Plant Sci. 10, 1246. 10.3389/fpls.2019.01246 31681363 PMC6805966

[B26] Ding T. Melcher U. (2016). Influences of plant species, season and location on leaf endophytic bacterial communities of non-cultivated plants. PloS One 11, e0150895. 10.1371/journal.pone.0150895 26974817 PMC4790846

[B27] Dissanayake A. J. Purahong W. Wubet T. Hyde K. D. Zhang W. Xu H. (2018). Direct comparison of culture-dependent and culture-independent molecular approaches reveal the diversity of fungal endophytic communities in stems of grapevine (Vitis vinifera). Fungal Divers. 90, 85–107. 10.1007/s13225-018-0399-3

[B28] Doebley J. F Gaut B. S. Smith B. D. (2006). The molecular genetics of crop domestication. Cell 127, 1309–1321. 10.1016/j.cell.2006.12.006 17190597

[B29] Durand A. Maillard F. Alvarez-Lopez V. Guinchard S. Bertheau C. Valot B. (2018). Bacterial diversity associated with poplar trees grown on a Hg-contaminated site: Community characterization and isolation of Hg-resistant plant growth-promoting bacteria. Sci. Total Environ. 622–623, 1165–1177. 10.1016/j.scitotenv.2017.12.069 29890585

[B30] Edwards J. Johnson C. Santos-Medellín C. Lurie E. Podishetty N. K. Bhatnagar S. (2015). Structure, variation, and assembly of the root-associated microbiomes of rice. Proc. Natl. Acad. Sci. 112, E911–E920. 10.1073/pnas.1414592112 25605935 PMC4345613

[B31] Ercolani G. L. (1991). Distribution of epiphytic bacteria on olive leaves and the influence of leaf age and sampling time. Microb. Ecol. 21, 35–48. 10.1007/BF02539143 24194200

[B32] Fausto C. Mininni A. N. Sofo A. Crecchio C. Scagliola M. Dichio B. (2018). Olive orchard microbiome: characterisation of bacterial communities in soil-plant compartments and their comparison between sustainable and conventional soil management systems. Plant Ecol. Divers. 11, 597–610. 10.1080/17550874.2019.1596172

[B33] Foster Z. S. L. Sharpton T. J. Grünwald N. J. (2017). Metacoder: An R package for visualization and manipulation of community taxonomic diversity data. PloS Comput. Biol. 13, e1005404. 10.1371/journal.pcbi.1005404 28222096 PMC5340466

[B34] Gagliardi J. V. Buyer J. S. Angle J. S. Russek-Cohen E. (2001). Structural and functional analysis of whole-soil microbial communities for risk and efficacy testing following microbial inoculation of wheat roots in diverse soils. Soil Biol. Biochem. 33, 25–40. 10.1016/S0038-0717(00)00110-3

[B35] Gardner J. M. Feldman A. W. Zablotowicz R. M. (1982). Identity and behavior of xylem-residing bacteria in rough lemon roots of Florida citrus trees. Appl. Environ. Microbiol. 43, 1335–1342. 16346030 10.1128/aem.43.6.1335-1342.1982 PMC244237

[B36] Ginnan N. A. Dang T. Bodaghi S. Ruegger P. M. Peacock B. B. McCollum G. (2018). Bacterial and fungal next generation sequencing datasets and metadata from citrus infected with ‘Candidatus Liberibacter asiaticus.'. Phytobiomes J. 2, 64–70. 10.1094/PBIOMES-08-17-0032-A

[B37] Gómez A. J. Infante-Amate J. De Molina G. M. Vanwalleghem T. Taguas V. E. Lorite I. (2014). Olive cultivation, its impact on soil erosion and its progression into yield impacts in Southern Spain in the past as a key to a future of increasing climate uncertainty. Agriculture 4, 170–198. 10.3390/agriculture4020170

[B38] Gómez-Lama Cabanás C. Legarda G. Ruano-Rosa D. Pizarro-Tobías P. Valverde-Corredor A. Niqui J. L. (2018). Indigenous *Pseudomonas* spp. Strains from the Olive (*Olea europaea* L.) Rhizosphere as Effective Biocontrol Agents against Verticillium dahliae: From the Host Roots to the Bacterial Genomes. Front. Microbiol. 9, 277. 10.3389/fmicb.2018.00277 29527195 PMC5829093

[B39] Hamedi J. Mohammadipanah F. (2015). Biotechnological application and taxonomical distribution of plant growth promoting actinobacteria. J. Ind. Microbiol. Biotechnol. 42, 157–171. 10.1007/s10295-014-1537-x 25410828

[B40] Iniesta F. Testi L. Orgaz F. Villalobos F. J. (2009). The effects of regulated and continuous deficit irrigation on the water use, growth and yield of olive trees. Europ. J. Agron. 30, 258–265. 10.1016/j.eja.2008.12.004

[B41] Izhaki I. Fridman S. Gerchman Y. Halpern M. (2013). Variability of bacterial community composition on leaves between and within plant species. Curr. Microbiol. 66, 227–235. 10.1007/s00284-012-0261-x 23143286

[B42] Jackson C. R. Randolph K. C. Osborn S. L. Tyler H. L. (2013). Culture dependent and independent analysis of bacterial communities associated with commercial salad leaf vegetables. BMC Microbiol. 13, 274. 10.1186/1471-2180-13-274 24289725 PMC4219373

[B43] Jiménez-Díaz R. M. Cirulli M. Bubici G. del Mar Jiménez-Gasco M. Antoniou P. P. Tjamos E. C. (2011). Verticillium wilt, a major threat to olive production: current status and future prospects for its management. Plant Dis. 96, 304–329. 10.1094/PDIS-06-11-0496 30727142

[B44] Khan A. L. Waqas M. Kang S.-M. Al-Harrasi A. Hussain J. Al-Rawahi A. (2014). Bacterial endophyte Sphingomonas sp. LK11 produces gibberellins and IAA and promotes tomato plant growth. J. Microbiol. 52, 689–695. 10.1007/s12275-014-4002-7 24994010

[B45] Kim H. Nishiyama M. Kunito T. Senoo K. Kawahara K. Murakami K. (1998). High population of Sphingomonas species on plant surface. J. Appl. Microbiol. 85, 731–736. 10.1111/j.1365-2672.1998.00586.x

[B46] Kumar P. Dubey R. C. Maheshwari D. K. (2012). Bacillus strains isolated from rhizosphere showed plant growth promoting and antagonistic activity against phytopathogens. Microbiol. Res. 167, 493–499. 10.1016/j.micres.2012.05.002 22677517

[B47] Lacava P. T. Li W. Araujo W. L. Azevedo J. L. Hartung J. S. (2007). The endophyte Curtobacterium flaccumfaciens reduces symptoms caused by Xylella fastidiosa in Catharanthus roseus. J. Microbiol. 45, 388–393. 17978797

[B48] Landa B. B. Pérez A. G. Luaces P. Montes-Borrego M. Navas-Cortés J. A. Sanz C. (2019). Insights into the effect of verticillium dahliae defoliating-pathotype infection on the content of phenolic and volatile compounds related to the sensory properties of virgin olive oil. Front. Plant Sci. 10, 232. 10.3389/fpls.2019.00232. 30891053 PMC6413673

[B49] Landa B. B. Castillo A. I. Giampetruzzi A. Kahn A. Román-Écija M. Velasco-Amo M. P. (2020). Emergence of a plant pathogen in Europe associated with multiple intercontinental introductions. Appl. Environ. Microbiol. 86, In press. 10.1128/AEM.01521-19. PMC6974645 31704683

[B50] Lavee S. (1990). “Aims, methods, and advances in breeding of new olive (*Olea europaea* L.) cultivars,” in Acta Horticulturae (Leuven, Belgium: International Society for Horticultural Science (ISHS)), 23–36. 10.17660/ActaHortic.1990.286.1

[B51] McCully M. E. (2001). Niches for bacterial endophytes in crop plants: a plant biologist's view. Funct. Plant Biol. 28, 983–990. 10.1071/PP01101

[B52] Mendes R. Pizzirani-Kleiner A. A. Araujo W. L. Raaijmakers J. M. (2007). Diversity of cultivated endophytic bacteria from sugarcane: genetic and biochemical characterization of *Burkholderia cepacia* Complex Isolates. Appl. Environ. Microbiol. 73, 7259–7267. 10.1128/AEM.01222-07 17905875 PMC2168197

[B53] Mendes R. Kruijt M. de Bruijn I. Dekkers E. van der Voort M. Schneider J. H. M. (2011). Deciphering the rhizosphere microbiome for disease-suppressive bacteria. Science *(80-.)**** 332, 1097–1100. 10.1126/science.1203980. 21551032

[B54] Mercado-Blanco J. Rodríguez-Jurado D. Hervás A. (2004). Suppression of Verticillium wilt in olive planting stocks by root-associated fluorescent Pseudomonas spp. Biol. Control 30, 474–486. 10.1016/j.biocontrol.2004.02.002

[B55] Montes-Borrego M. Navas-Cortés J. A. Landa B. B. (2013). Linking microbial functional diversity of olive rhizosphere soil to management systems in commercial orchards in southern Spain. Agric. Ecosyst. Environ. 181, 169–178. 10.1016/j.agee.2013.09.021

[B56] Montes-Borrego M. Metsis M. Landa B. B. (2014). Arbuscular mycorhizal fungi associated with the olive crop across the Andalusian landscape: Factors driving community differentiation. PloS One 9 (5) e96397. 10.1371/journal.pone.0096397 24797669 PMC4010464

[B57] Müller H. Berg C. Landa B. B. Auerbach A. Moissl-Eichinger C. Berg G. (2015). Plant genotype-specific archaeal and bacterial endophytes but similar Bacillus antagonists colonize Mediterranean olive trees. Front. Microbiol. 6, 138. 10.3389/fmicb.2015.00138 25784898 PMC4347506

[B58] Ondov B. D. Bergman N. H. Phillippy A. M. (2011). Interactive metagenomic visualization in a Web browser. BMC Bioinform. 12, 385. 10.1186/1471-2105-12-385 PMC3190407 21961884

[B59] Palaniyandi S. A. Yang S. H. Zhang L. Suh J.-W. (2013). Effects of actinobacteria on plant disease suppression and growth promotion. Appl. Microbiol. Biotechnol. 97, 9621–9636. 10.1007/s00253-013-5206-1 24092003

[B60] Peiffer J. A. Spor A. Koren O. Jin Z. Tringe S. G. Dangl J. L. (2013). Diversity and heritability of the maize rhizosphere microbiome under field conditions. Proc. Natl. Acad. Sci. 110, 6548–6553. 10.1073/pnas.1302837110 23576752 PMC3631645

[B61] Pérez-Jaramillo J. E. Carrión V. J. Bosse M. Ferrão L. F. V. de Hollander M. Garcia A. A. F. (2017). Linking rhizosphere microbiome composition of wild and domesticated Phaseolus vulgaris to genotypic and root phenotypic traits. ISME J. 11, 2244–2257. 10.1038/ismej.2017.85 28585939 PMC5607367

[B62] Prieto P. Schilirò E. Maldonado-González M. M. Valderrama R. Barroso-Albarracín J. B. Mercado-Blanco J. (2011). Root hairs play a key role in the endophytic colonization of olive roots by pseudomonas spp. with biocontrol activity. Microb. Ecol. 62, 435–445. 10.1007/s00248-011-9827-6 21347721 PMC3155037

[B63] Qin S. Li J. Chen H.-H. Zhao G.-Z. Zhu W.-Y. Jiang C.-L. (2009). Isolation, diversity, and antimicrobial activity of rare actinobacteria from medicinal plants of tropical rain forests in Xishuangbanna, China. Appl. Environ. Microbiol. 75, 6176–6186. 10.1128/AEM.01034-09 19648362 PMC2753051

[B64] Rajendran P. Sundaram S. P. Kumutha K. (2009). *In vitro* biocontrol activity of methylobacterium extorquens against fungal pathogens. Int. J. Plant Prot. 2, 59–62. 10.13140/2.1.3086.0163

[B65] Reasoner D. J. Geldreich E. E. (1985). A new medium for the enumeration and subculture of bacteria from potable water. Appl. Environ. Microbiol. 49, 1–7. Available at: http://aem.asm.org/content/49/1/1.abstract. 10.1128/AEM.49.1.1-7.1985 3883894 PMC238333

[B66] Rey P. J. (2011). Preserving frugivorous birds in agro-ecosystems: lessons from Spanish olive orchards. J. Appl. Ecol. 48, 228–237. 10.1111/j.1365-2664.2010.01902.x

[B68] Saponari M. Giampetruzzi A. Loconsole G. Boscia D. Saldarelli P. (2018). Xylella fastidiosa in Olive in Apulia: Where We Stand. Phytopathology 109, 175–186. 10.1094/PHYTO-08-18-0319-FI 30376439

[B69] Sofo A. Mininni A. N. Fausto C. Scagliola M. Crecchio C. Xiloyannis C. (2019). primers. Sci. Total Environ. 658, 763–767. 10.1016/j.scitotenv.2018.12.264 30583171

[B70] Tuo L. Pan Z. Li F.-N. Lou I. Guo M. Lee S. M.-Y. (2016). Friedmanniella endophytica sp. nov., an endophytic actinobacterium isolated from bark of Kandelia candel. Int. J. Syst. Evol. Microbiol. 66, 3057–3062. 10.1099/ijsem.0.001146 27169592

[B71] Turner T. R. James E. K. Poole P. S. (2013). The plant microbiome. Genome Biol. 14, 209. 10.1186/gb-2013-14-6-209 23805896 PMC3706808

[B72] Ulrich K. Ulrich A. Ewald D. (2008). Diversity of endophytic bacterial communities in poplar grown under field conditions. FEMS Microbiol. Ecol. 63, 169–180. 10.1111/j.1574-6941.2007.00419.x 18199082

[B73] Uroz S. Buée M. Murat C. Frey-Klett P. Martin F. (2010). Pyrosequencing reveals a contrasted bacterial diversity between oak rhizosphere and surrounding soil. Environ. Microbiol. Rep. 2, 281–288. 10.1111/j.1758-2229.2009.00117.x 23766079

[B74] Van Aken B. M Peres C. Doty S. Yoon J. M. Schnoor J. (2004). Methylobacterium populi sp. nov., a novel aerobic, pink-pigmented, facultative methylotrophic, methane-utilizing bacterium isolated from poplar trees (Populus deltoidesxnigra DN34). Int. J. Syst. Evol. Microbiol. 54, 1191–1196. 10.1099/ijs.0.02796-0 15280290

[B75] Vandenkoornhuyse P. Quaiser A. Duhamel M. Le Van A. Dufresne A. (2015). The importance of the microbiome of the plant holobiont. New Phytol. 206, 1196–1206. 10.1111/nph.13312 25655016

[B76] Wang Q. Garrity G. M. Tiedje J. M. Cole J. R. (2007). Naïve bayesian classifier for rapid assignment of rRNA sequences into the new bacterial taxonomy. Appl. Environ. Microbiol. 73, 5261–5267. 10.1128/AEM.00062-07. 17586664 PMC1950982

[B77] Weisburg W. G. Barns S. M. Pelletier D. A. Lane D. J. (1991). 16S ribosomal DNA amplification for phylogenetic study. J. Bacteriol. 173, 697–703. 10.1128/jb.173.2.697-703.1991 1987160 PMC207061

[B78] Wells J. M. Raju B. C. Nyland G. Lowe S. K. (1981). Medium for isolation and growth of bacteria associated with plum leaf scald and phony peach diseases. Appl. Environ. Microbiol. 42, 357–363. Available at: http://aem.asm.org/content/42/2/357.abstract. 16345835 10.1128/aem.42.2.357-363.1981 PMC244013

[B79] White D. C. Sutton S. D. Ringelberg D. B. (1996). The genus Sphingomonas: physiology and ecology. Curr. Opin. Biotechnol. 7, 301–306. 10.1016/S0958-1669(96)80034-6 8785434

[B80] Yang R. Liu P. Ye W. (2017). Illumina-based analysis of endophytic bacterial diversity of tree peony (Paeonia Sect. Moutan) roots and leaves. Braz. J. Microbiol. 48, 695–705. 10.1016/j.bjm.2017.02.009 28606427 PMC5628320

