# Supplementary material for: Culture-Dependent and Culture-Independent Characterization of the Olive Xylem Microbiota: Effect of Sap Extraction Methods
Source: Front Plant Sci. 2020 Jan 21;10:1708. doi: 10.3389/fpls.2019.01708 (PMC6988092; doi:10.3389/fpls.2019.01708)
Supplement: Figure S1 — Comparison of rarefaction curves of observed OTUs present in the xylem sap of ‘Picual’ (Pic), ‘Arbequina’ (Arb), and ‘Acebuche’ (Ace) olive genotypes extracted with the Scholander chamber (SCh) or from woody chips macerates (WC). Error bars represent standard derivation of three independent tree replicates. Data were rarified to 1,234 sequences. [file DataSheet_1.zip › Image 3.ZIP!/Figure S3. Abundances culture-dependent approach.html]

Javascript must be enabled to view this page.

magnitude
magnitudeUnassigned

cultivables

99.99

46.37

45.99

39.47

34.49

0.77

0.77

0.38

18.77

11.88

1.92

0.38

0.38

1.53

1.53

1.92

1.92

1.15

0.77

0.38

1.15

1.15

1.15

1.15

0.77

0.77

0.38

0.38

3.07

3.07

1.53

0.77

0.77

1.15

1.15

0.77

0.38

0.38

0.38

0.38

0.38

0.38

0.38

0.38

0.38

0.38

19.15

19.15

0.38

0.38

0.38

18.77

1.92

1.92

3.45

1.92

1.53

0.38

0.38

13.02

12.64

0.38

34.09

32.18

0.38

0.38

0.38

14.94

14.94

0.38

14.56

16.86

16.86

16.86

0.38

0.38

0.38

0.38

1.53

1.53

1.53

1.53
